# Myeloid PD‐1 Regulates Astrocyte Development and Leads to Active Behaviours

**DOI:** 10.1111/cpr.70082

**Published:** 2025-06-29

**Authors:** Jie Qin, Chong Wang, Sihan Li, Yanyan Wang, Tingting He, Jianwei Jiao, Fen Ji

**Affiliations:** ^1^ State Key Laboratory of Organ Regeneration and Reconstruction Institute of Zoology, Chinese Academy of Sciences Beijing China; ^2^ University of Chinese Academy of Sciences Beijing China; ^3^ Beijing Institute for Stem Cell and Regenerative Medicine Beijing China; ^4^ Sino‐Danish College at University of Chinese Academy of Sciences Beijing China; ^5^ University of Science and Technology of China Hefei Anhui China

**Keywords:** astrocyte, myeloid cell, neural stem cells (NSCs), PD‐1, proliferation and differentiation

## Abstract

During early brain development, the nervous system evolves as cells connect to form a unique neural network, with communication between cell populations vital for neurological balance. This study investigates how the loss of PD‐1 in myeloid cells disrupts nervous system development. Specific ablation of PD‐1 affects myeloid cell proliferation and classification. As astrogenesis begins, astrocyte proliferation ceases, continuous astrocyte proliferation is observed. Immunofluorescence staining revealed high expression of astrocyte‐related genes in PD‐1^f/f; LysM‐Cre^ mice, which also exhibited more extroverted behaviour. Additionally, the absence of PD‐1 enhances CXCL1 expression through the NF‐κB pathway, promoting astrocyte proliferation by interacting with CXCR2. These findings underscore PD‐1's regulatory role in myeloid cells and its implications for the myeloid‐brain axis.

## Graphical abstract

During early neurodevelopment, PD‐1 ablation in myeloid cells influences myeloid proliferation/differentiation and causes sustained astrocyte overproliferation. PD‐1^f/f; LysM‐Cre^ mice showed elevated astrocyte gene expression and behavioural changes. Mechanistically, PD‐1 deletion activates NF‐κB‐mediated CXCL1 secretion, driving astrocyte proliferation via CXCR2, revealing PD‐1's critical role in myeloid‐astrocyte crosstalk.

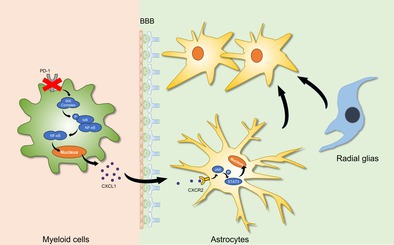

## 1 Introduction

Brain development is governed by a complex interplay of intrinsic and extrinsic factors, with corticogenesis exhibiting conserved mechanisms across mammalian species [1]. Neurogenesis is tightly regulated through precise cell–cell interactions [2], and perturbations in neuronal development have been implicated in various neurodevelopmental disorders [3]. Notably, immune mediators such as cytokines (e.g., IL‐6, TNF‐α) and immune cells exert differential effects on neurogenesis and synaptic pruning [4, 5]. Immune dysregulation has been shown to disrupt neurodevelopmental processes and alter behavioural outcomes [6, 7], whereas a balanced inflammatory response plays a critical role in mediating neurorepair while mitigating neurotoxicity [8, 9].

The myeloid system is derived from the haematopoietic system, which is regenerated and widespread throughout the body. Since the brain primarily develops during the early stages of life and focuses on repair in later stages, the regenerative system plays a crucial role in regulating the non‐regenerative system [10, 11]. Programmed cell death protein 1 (PD‐1), as an important immunosuppressive molecule, has an important role in the whole immune system. PD‐1 is present on the surface and binds to PD‐L1, which delivers a suppressive immune response, like suppression of T‐cell, inducing apoptosis of T‐cell and releasing IL‐10 [12]. Myeloid cells are derived from myeloid progenitor cells in the bone marrow. Targeted deletion of PD‐1 in myeloid cells in mice was more effective at suppressing tumour growth than deletion of PD‐1 in T cells, suggesting that myeloid‐specific PD‐1 knockout influences myeloid cell fate determination [13]. The use of PD‐1 blockers results in a reduction in the concentrations of tyrosine and tryptophan in the patient's blood, which further contributes to deficiencies of dopamine (DA) and serotonin (5‐hydroxytryptophan, 5‐HT), leading to the development of negative emotions such as depression and fear [14]. Endogenous immune responses are harnessed in immunotherapy and targeted drug therapies to induce tumour regression; however, these treatments may also result in abnormal T‐cell metabolism, potentially impacting the nervous system. The myeloid system is closely related to the immune system and frequently assumes a secondary role in promoting or suppressing the inflammatory response. PD‐1‐positive immune cells, activated in the central nervous system (CNS) under pathological conditions, travel through cervical lymph nodes into the brain [15].

The role of PD‐1 in myeloid cells during physiological brain development remains largely unexplored. Here, we demonstrate that PD‐1 modulates myeloid cell proliferation, subtype differentiation, and astrocyte generation through a CXCL1‐dependent mechanism. At the molecular level, PD‐1 activation triggers NF‐κB signalling in myeloid cells, leading to CXCL1 secretion, which subsequently binds to CXCR2 on astrocytes to facilitate astrogenesis. These findings uncover a novel PD‐1‐dependent neuro‐immune signalling axis critical for brain development.

## 2 Material and Methods

### 2.1 Experimental Animals and Cells

The PD‐1 floxed mice used in this experiment were purchased from Shanghai Southern Model Biotechnology Co. The LysM‐Cre tool mice were purchased from Jackson (USA) Laboratories. ICR strain pregnant mice and wild‐type C57BL/6J strain mice were purchased from Beijing Vitalriver Technology Co. The mice used in the experiments were kept and bred, and the animal experiments were performed in strict compliance with the relevant regulations for laboratory animals.

The primary neural stem cells used in this experiment were obtained from the cerebral cortex of fetal mice at an early period.

Mice used in this study were constructed by CRISPR‐CAS9 technology to insert the LoxP sequence at both ends of the exon of *Pdcd1*. Pd1 mice were generated by mating with LysM‐Cre mice (CRE recombinase specifically expressed in myeloid cells) to obtain PD‐1^f/f; LysM‐Cre^ mice and PD‐1^f/f^ mice.

### 2.2 Flow Cytometry

Fetal liver cells were dissociated using Papain, filtered through 40‐μm strainers, and washed three times in PBS. Cells were fixed in 4% PFA (30 min, RT), washed, and surface‐stained with flow antibodies (1:1000, 4°C, 25–30 min) (Table S3). After washing, 1 × 10^6^ cells were resuspended in PBS+2.5% FBS and analysed using a BD FACS Aria III.

### 2.3 Quantitative Real‐Time PCR

Total RNA was extracted using Trizol (Invitrogen) with RNase‐free reagents. Samples were lysed in 1 mL Trizol, mixed with 200 μL trichloromethane, shaken vigorously, and centrifuged (13,000 rpm, 15 min, 4°C). The aqueous phase was collected, mixed with isopropanol, and centrifuged again. RNA pellets were washed with 75% ethanol, air‐dried, and dissolved in RNase‐free ddH_2_O. Purity (OD260/280: 1.8–2.0) was checked before storage at −80°C.

The cDNA was synthesised using a FastQuant RT Kit (Tiangen). qPCR was performed on a 7500 system (Applied Biosystems) with SYBR master mix (Tiangen) and gene‐specific primers (Table S4). β‐Actin served as the endogenous control. Reactions were run in triplicate.

### 2.4 Western Blotting

Cells or tissues were lysed in RIPA buffer (Solarbio) with protease inhibitors, then centrifuged (12,000 rpm, 15 min). Proteins were denatured in loading buffer (10 min, boiling), separated by SDS‐PAGE, and transferred to NC/PVDF membranes. After blocking (5% milk in PBST, 1 h, RT), membranes were incubated with primary antibodies in 4°C, overnight (Table S1), washed, and probed with secondary antibodies in RT for 1 h (Table S2). Detection was performed using an Odyssey fluorescent scanner.

### 2.5 Immunostaining

Fixed brains were sectioned (20 μm) and post‐fixed in 4% PFA (30 min). After washing (1% and 0.1% PBST, 3 × 10 min), sections were blocked (5% BSA in PBST, 1 h, RT) and incubated with primary antibodies at 4°C overnight (Table S1). Following PBST washes, samples were incubated with fluorescent secondary antibodies at RT for 1 h (Table S2), washed again, and imaged.

### 2.6 Confocal Imaging and Statistical Analysis

All images were obtained by a Zeiss LSM880 and processed with Photoshop CC 2020 (Adobe). The fluorescence density was calculated with Zeiss ZEN 2012 blue version. Quantitative data are presented as the means ± SEMs. The data were analysed by an unpaired t‐test. For multiple comparisons, the data were analysed by ANOVA. *p < 0.05, **p < 0.01, and ***p < 0.001. All statistical analyses were performed using GraphPad Prism 9.

### 2.7 Three‐Chamber Social Test

A test mouse was placed in the central chamber of a three‐chamber apparatus, with an unfamiliar mouse under a wire cage in the left chamber and an empty cage in the right. Interaction time (sniffing/chamber occupancy) was recorded for 10 min. Immediately following, the same apparatus was used with the familiar mouse in the left chamber and a novel mouse in the right. The test mouse's interactions with both were recorded for 10 min.

### 2.8 Elevated Plus Maze

The EPM consists of two open and two closed arms connected by a central area. Mice were allowed to explore freely for 5 min while being recorded. Anxiety‐like behaviour was assessed by calculating the percentage of entries and time spent in open versus closed arms.

### 2.9 Open Field Test

The procedure of observing the activity of the mice by placing them in a closed flat area, which was divided into center and outside. Usually, within 5 min animals cross the square of the count to measure their activities.

### 2.10 Novel Object Recognition Test

Mice were first familiarised with two identical objects for 10 min. Later, one object was replaced with a novel one, and their exploratory behaviour was recorded for 10 min to assess recognition memory.

### 2.11 Y‐Maze Test

The apparatus had three arms (120° apart). Initially, one arm was blocked (novel arm) during 5 min exploration. In the test phase, all arms were opened, and mouse activity was recorded for 5 min.

### 2.12 Co‐Immunoprecipitation

Forty‐eight hours post‐transfection, cells were washed with ice‐cold PBS and lysed in RIPA buffer (500 μL, 1% PMSF/protease inhibitor) using scraping. Lysates were sonicated (15% power, 5 s‐on/10s‐off, 3 cycles) on ice, centrifuged (12,000 rpm, 4°C, 15–20 min), and supernatants collected. For co‐IP, lysates were incubated with antibody‐bound magnetic beads (4°C, overnight). Beads were washed 3–4 times with buffer, proteins eluted in SDS loading buffer (95°C, 5 min), and analysed via SDS‐PAGE/Western blot.

### 2.13 Co‐Culture

The Cytoselect 24‐well system enabled direct contact between neural stem cells (NSCs) and myeloid cells. NSCs (E16/P3) were plated on lower‐layer slices, while flow‐sorted myeloid cells were added to the upper layer. Cultures were maintained in NSC (DMEM/FBS/PS) and myeloid (RPMI1640/FBS) media, with a higher lower‐medium level. After 3 days, cells were stained and imaged.

### 2.14 RNA‐Seq Analyses

Total RNA was extracted from P2 fetal livers of wild‐type and PD‐1 knockout mice. Sequencing (Illumina HiSeq 2500) was performed by Annoroad Genomics after QC (Agilent 2100 RNA Nano 6000). Gene expression was quantified as RPKM, with differentially expressed genes defined as |log2FC| ≥ 1 and *q* < 0.05. Enriched GO terms (*q* < 0.05) and key downstream genes were identified.

## 3 Results

### 3.1 PD‐1 Is Expressed in Myeloid Cells and Correlated With Brain Development

**FIGURE 1:**
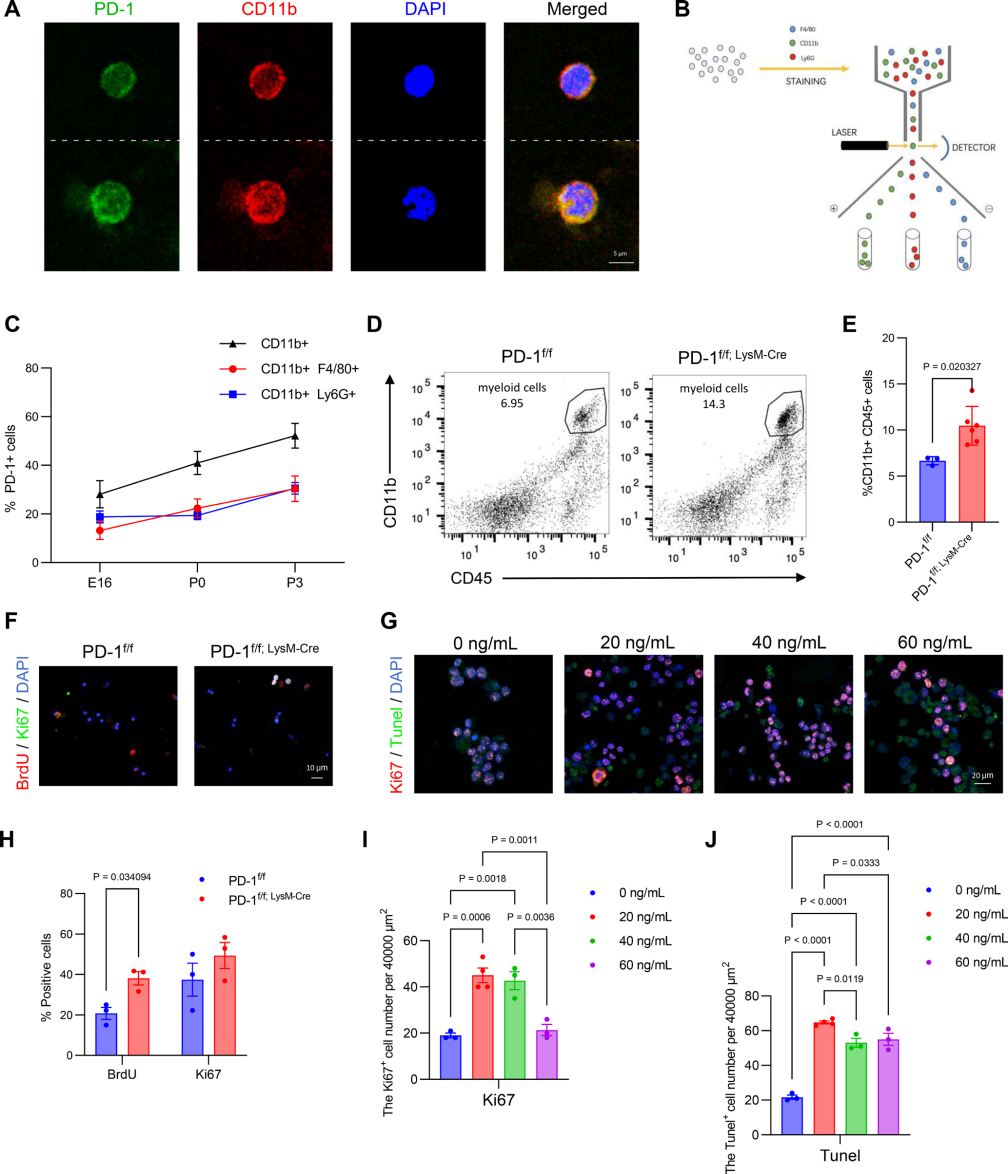
Deletion of targeted deletion of PD‐1 increased proliferation in myeloid cells. (A) Confocal immunofluorescence image of PD‐1 and CD11b in myeloid cells. (B) Schematic overview of cytometry flow used to sort myeloid cells. (C) Kinetics of PD‐1 up‐regulation on CD11b^+^Ly6G^+^, CD11b^+^F4/80^+^, and CD11b^+^ cells, sorting from fetal liver (*n* = 3). (D) Representative flow cytometry plots depicting the proportion of CD11b^+^CD45^+^ myeloid cells in the fetal liver. (E) Quantification of the increase in the number of myeloid cells in PD‐1^f/f; LysM‐Cre^ mice (*n* ≥ 3). (F) Confocal immunofluorescence images showed BrdU‐labelled and Ki67‐labelled myeloid cells. (G) Confocal immunofluorescence images displayed Ki67‐ and TUNEL‐labelled K562 cell lines treated with varying concentrations of anti‐PD‐1. (H) Quantification of the increase in the number of BrdU^+^ myeloid cells in PD‐1^f/f; LysM‐Cre^ mice (*n* = 3). (I) Quantification of the Ki67^+^ cell number per 40,000 μm^2^ (*n* ≥ 3). (J) Quantification of the Tunel^+^ cell number per 40,000 μm^2^ (*n* ≥ 3). Data are presented as mean ± SEM. One‐way ANOVA and multiple t‐tests and nonparametric tests.

PD‐1, an immunoinhibitory receptor well‐characterised in adaptive immunity [16], was observed to co‐localise with CD11b^+^ myeloid cell populations in the developing brain (Figure 1A). Quantitative analysis of myeloid subpopulations, including CD11b^+^Ly6G^+^ granulocytes and CD11b^+^F4/80^+^ macrophages, demonstrated progressively upregulated PD‐1 expression from embryonic day 16 (E16) through postnatal day 3 (P3) (Figure 1B,C), indicative of stage‐specific regulation during corticogenesis. These observations provide compelling rationale for further investigation into PD‐1's potential role in modulating neurodevelopmental immune processes.

### 3.2 The Deletion of PD‐1 in Myeloid Cells Leads to Proliferation During Brain Development

To investigate the role of PD‐1 in myeloid cells, we generated conditional knockout mice (PD‐1^f/f; LysM‐Cre^) exhibiting significant PD‐1 reduction specifically in myeloid lineages, as confirmed by qPCR and Western blot analyses (Figure S1A–C,F). Although LysM‐Cre has been reported to target neuronal and microglial populations [17], immunohistochemical analysis revealed minimal non‐specific recombination in these cell types (Figure S1D,E,G–J). PD‐1 ablation resulted in a marked expansion of CD11b^+^CD45^+^ myeloid cells (Figure 1D,E) accompanied by enhanced proliferative capacity, as evidenced by BrdU incorporation assays (Figure 1F,H). In vitro experiments demonstrated a dose‐dependent effect of PD‐1 blockade, with low‐dose anti‐PD‐1 antibody promoting K‐562 cell proliferation while high‐dose treatment induced apoptosis (Figure 1G,I,J).

**FIGURE 2:**
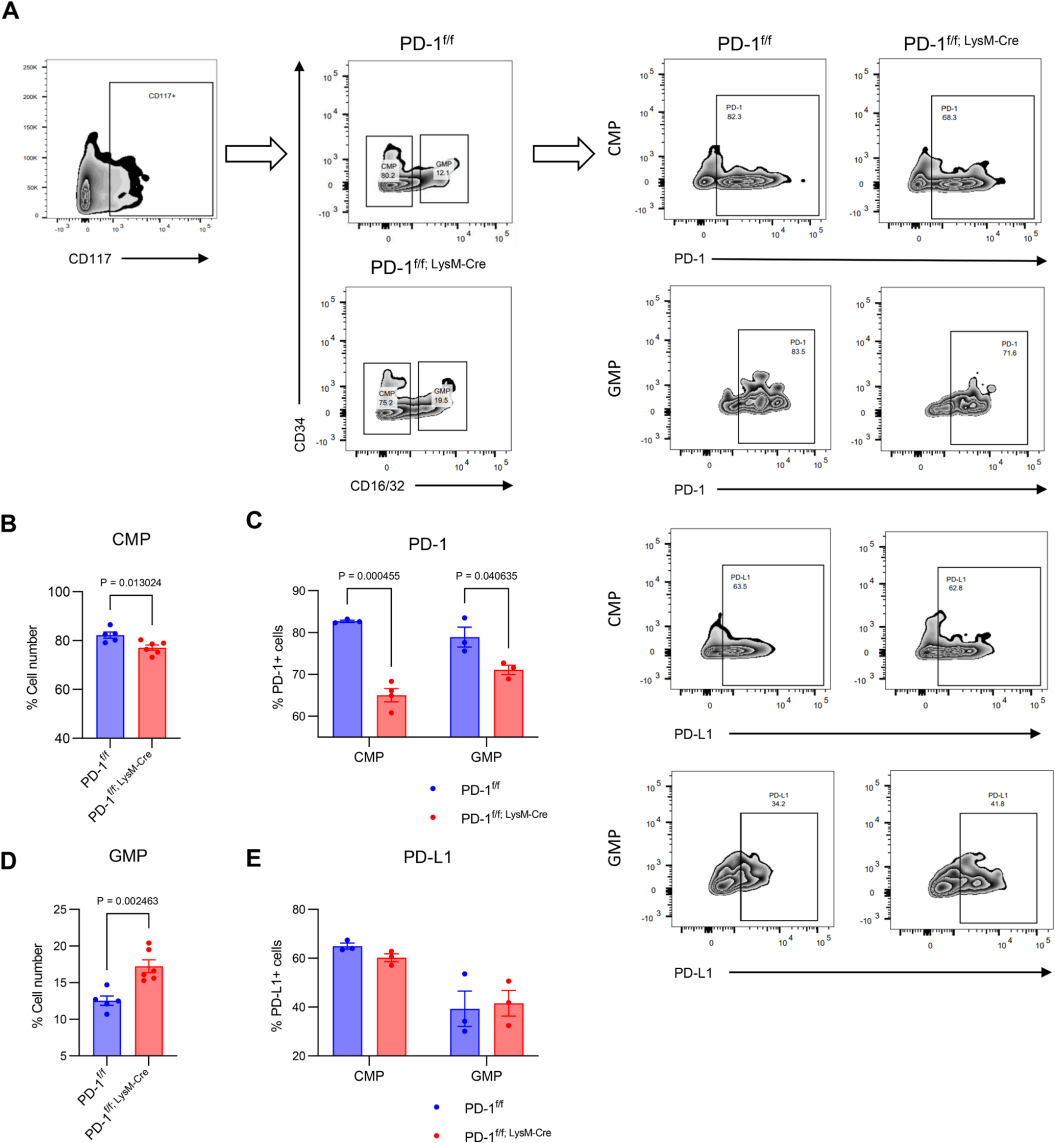
Effect of PD‐1 targeted myeloid deletion on myeloid progenitors. (A) Representative plots of FACS analysis for PD‐1 and PD‐L1 on CMP and GMP of PD‐1^f/f^ mice and PD‐1^f/f; LysM‐Cre^ mice. (B and D) Quantification of the decrease in the number of CMP and the increase in the number of GMP in PD‐1^f/f; LysM‐Cre^ mice (*n* ≥ 5). (C and E) Quantification of the number of PD‐1 and PD‐L1 in CMP and GMP (*n* = 3). Data are presented as mean ± SEM. Multiple t‐tests and nonparametric tests.

Genetic deletion of PD‐1 significantly altered myeloid subset distribution, characterised by increased CD11b^+^Ly6C^+^ monocytes but decreased CD11b^+^Ly6G^+^ granulocytes and CD11b^+^F4/80^+^ macrophages (Figure S2A,C,E), without modulating T‐cell‐associated cytokine production (Figure S2F). Haematopoietic progenitor analysis revealed that PD‐1^f/f; LysM‐Cre^ mice exhibited elevated granulocyte‐monocyte progenitors (GMPs) and reduced common myeloid progenitors (CMPs), with corresponding decreases in PD‐1 expression but unaltered PD‐L1 levels (Figure 2A–E). Flow cytometric sorting demonstrated monocyte predominance in isolated populations (Figure S2B,D), collectively suggesting that PD‐1 plays a critical role in regulating GMP differentiation fate decisions.

### 3.3 Specific Ablation of PD‐1 in Myeloid Cells Exhibit Anxiety‐Less Behaviours and Prefer to Explore Novel Objects and Areas, to Socialise With Strangers

**FIGURE 3:**
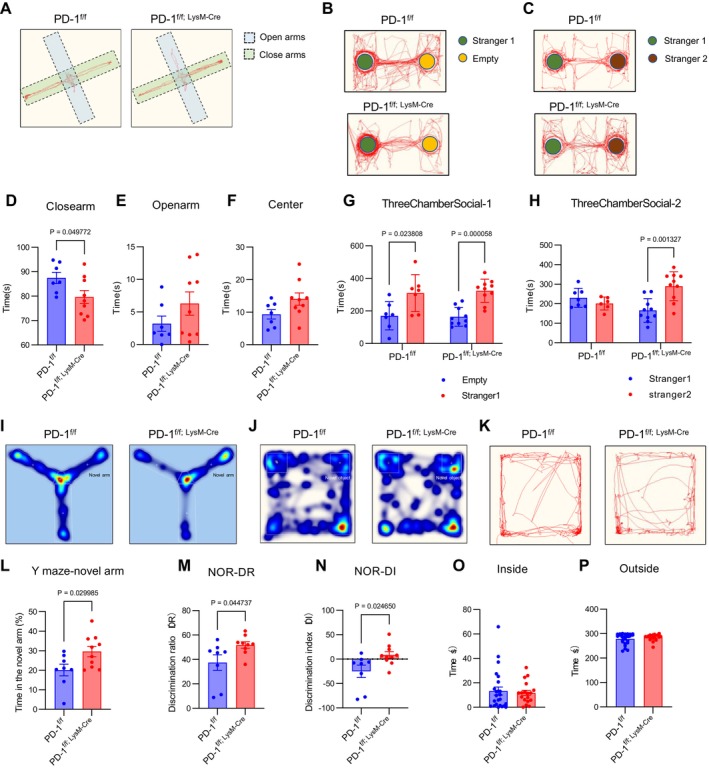
PD‐1^f/f; LysM‐Cre^ mice exhibited anxiety‐less behaviours and liked exploring novel objects and areas and socialising with strangers. (A) Representative trace plot for the high‐plus maze. (B and C) Representative trace plot illustrating the patterns of social affiliation and sociability. (D–F) The time spent in closed arms (D) and opened arms (E) and center (F) in the elevated plus‐maze test (*n* ≥ 7). (G and H) Social affiliation and sociability duration (G) in the sociability session and social memory and novelty duration (H) in the social novelty preference session in the three‐chamber social interaction test (*n* ≥ 7). (I) Representative trace plot for the Y‐maze. (J) Representative trace plot for the novel object recognition test. (K) Representative trace plot for the open field test. (L) PD‐1^f/f; LysM‐Cre^ mice spent significantly more time in the novel arm compared to controls (*n* ≥ 8). (M, N) The discrimination score and discrimination ratio were higher in PD‐1^f/f; LysM‐Cre^ mice in the novel object recognition test (*n* ≥ 8). (O and P) The time spent in the center area and outside (*n* ≥ 18). Data are presented as mean ± SEM. Multiple t‐tests and nonparametric tests.

Emerging evidence indicates that cranial bone marrow‐derived myeloid cells, including PD‐1^+^CD11b^+^ populations localised within meningeal tissues (Figure S1K), play a crucial role in modulating neurodevelopmental processes through intercellular signalling mechanisms [11, 18]. Behavioural characterisation of PD‐1^f/f; LysM‐Cre^ mice revealed a distinct neurobehavioral phenotype marked by reduced anxiety‐like behaviours, as demonstrated by significantly increased open‐arm exploration in the elevated plus maze (Figure 3A,D–F), coupled with enhanced sociability evidenced by a robust preference for novel conspecifics in the three‐chamber test (Figure 3B,C,G,H). These mice additionally exhibited improved cognitive performance, showing superior spatial working memory in Y‐maze testing (Figure 3I,L) and enhanced recognition memory in novel object recognition tests (discrimination index > 0.5; Figure 3J,M,N). Importantly, these behavioural modifications occurred independently of locomotor alterations, as open field testing revealed no significant differences in anxiety‐related or hyperactivity measures (Figure 3K,O,P). Collectively, these findings demonstrate that myeloid‐specific PD‐1 deletion produces a behavioural profile characterised by reduced anxiety‐like responses alongside heightened exploratory drive and social motivation, without affecting general motor function.

### 3.4 PD‐1 Mediates Astrogenesis During Central Nervous System Development

**FIGURE 4:**
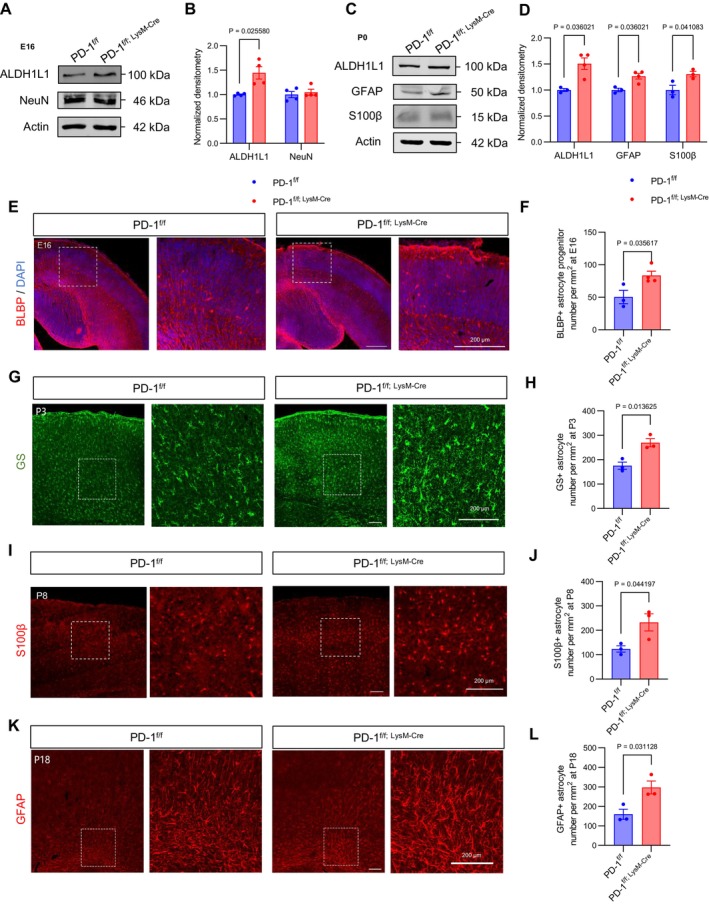
Specific ablation of PD‐1 in myeloid cells leads to persistent increased astrocyte production. (A) Western blot analysis of the expression levels of astrocyte marker ALDH1L1 and neuron marker NeuN. Actin was detected as loading control. (B) Statistics of the relative intensity of ALDH1L1 showing increased expression in PD‐1^f/f; LysM‐Cre^ mice and no change in NeuN (*n* = 4). (C) Western blot analysis of the expression levels of astrocyte markers ALDH1L1, GFAP, and S100β. Actin was detected as loading control. (D) Statistics of the relative intensity of ALDH1L1, GFAP, and S100β showing increased expression in PD‐1^f/f; LysM‐Cre^ mice (*n* ≥ 3). (E) Confocal immunofluorescence image showed BLBP^+^ progenitor astrocytes per square millimetre at E16 (Scale bars, 200 μm). (F) Quantification of the increase in the number of BLBP^+^ progenitor astrocytes in PD‐1^f/f; LysM‐Cre^ mice (*n* ≥ 3). (G) Confocal immunofluorescence image showed GS^+^ astrocytes per square millimetre at P3 (Scale bars, 200 μm). (H) Quantification of the increase in the number of GS^+^ progenitor astrocytes in PD‐1^f/f; LysM‐Cre^ mice (*n* = 3). (I) Confocal immunofluorescence image showed S100β^+^ astrocytes per square millimetre at P8 (Scale bars, 200 μm). (J) Quantification of the increase in the number of S100β^+^ astrocytes in PD‐1^f/f; LysM‐Cre^ mice (*n* = 3). (K) Confocal immunofluorescence image showed GFAP^+^ progenitor astrocytes per square millimetre at P18 (Scale bars, 200 μm). (L) Quantification of the increase in the number of GFAP^+^ astrocytes in PD‐1^f/f; LysM‐Cre^ mice (*n* = 3). Data are presented as mean ± SEM. Multiple t‐tests and nonparametric tests.

We focused our investigation on brain development in response to the observed behaviours in mice. To investigate myeloid‐specific PD‐1's role in brain development, we analysed cerebral cortices from PD‐1^f/f; LysM‐Cre^ and PD‐1^f/f^ mice at E16 and P0. Western blot revealed increased astrocyte marker aldehyde dehydrogenase 1 family member L1 (ALDH1L1) expression at E16, with unchanged neuronal marker neuronal nuclear protein (NeuN) expression (Figure 4A,B). By P0, elevated levels of ALDH1L1, glial fibrillary acidic protein (GFAP), and S100 calcium‐binding protein B (S100β) were observed (Figure 4C,D), while oligodendrocyte transcription factor 2‐positive (Olig2^+^) and Platelet derived growth factor receptor alpha‐positive (PDGFRα^+^) oligodendrocytes remained unaffected (Figure S3G–J), indicating selective astrocytic effects. Immunostaining demonstrated increased brain lipid‐binding protein‐positive (BLBP^+^) astrocyte progenitors at E16 (Figure 4E,F), followed by elevated glutamine synthetase‐positive (GS^+^) and GFAP^+^ astrocytes at P3 (Figures 4G,H, S3A,B). This astrocytic expansion persisted through P8 and P18, with higher S100β^+^ and GFAP^+^ cell counts (Figures 4I–L, S3C–F), suggesting sustained enhancement of astrogenesis. We co‐labelled proliferating astrocytes with GFAP and PH3 (Figure S4A,B). PD‐1^f/f; LysM‐Cre^ mice showed more GFAP^+^PH3^+^ cells than PD‐1^f/f^ mice, indicating increased astrocyte proliferation. These results indicated that specific PD‐1 ablation in myeloid cells not only affects the number of astrocytes but also enhances astrogenesis and persistently increases astrocyte production in the cerebral cortex during both the onset and long‐term stages.

Transwell co‐culture experiments confirmed myeloid‐derived factors directly influence astrocytes, increasing their numbers and altering morphology (Figure S4C–F). These findings demonstrate myeloid PD‐1 ablation specifically promotes astrocyte production and maturation throughout cortical development.

### 3.5 PD‐1 Promotes Proliferation and Regulates the Fate Determination of Radial Glial Cells

**FIGURE 5:**
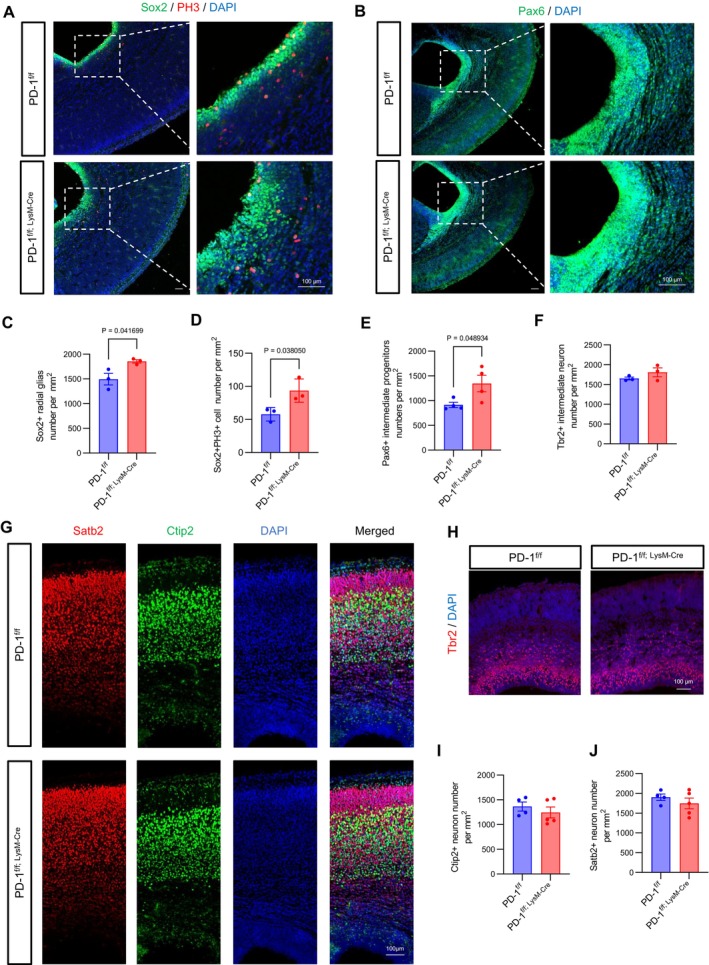
Specific ablation of PD‐1 in myeloid cells leads to increased proliferation of NSCs. (A) Confocal immunofluorescence image showed Sox2^+^ radial glia and PH3^+^ cells per square millimetre at E16 (Scale bars, 100 μm). (B) Confocal immunofluorescence image showed Pax6^+^ radial glia per square millimetre at E16 (Scale bars, 100 μm). (C) Quantification of the increase in the number of Sox2^+^ radial glia in PD‐1^f/f; LysM‐Cre^ mice (*n* = 3). (D) Quantification of the increase in the number of PH3^+^Sox2^+^ cells in PD‐1^f/f; LysM‐Cre^ mice (*n* = 3). (E) Quantification of the increase in the number of Pax6^+^ radial glia in PD‐1^f/f; LysM‐Cre^ mice (*n* = 4). (F) Quantification of unchanged the number of Tbr2^+^ intermediate progenitor neurons in PD‐1^f/f; LysM‐Cre^ mice (*n* = 3). (G) Confocal immunofluorescence image showed Satb2^+^ and Ctip2^+^ neurons per square millimetre at E16 (Scale bars, 100 μm). (H) Confocal immunofluorescence image showed Tbr2^+^ intermediate progenitor neuron per square millimetre at E16 (Scale bars, 100 μm). (I and J) Quantification of unchanged the number of Satb2^+^ and Ctip2^+^ neurons in PD‐1^f/f; LysM‐Cre^ mice (*n* ≥ 4). Data are presented as mean ± SEM. Multiple t‐tests and nonparametric tests.

To assess whether myeloid PD‐1 ablation affects astrocyte development from neural stem cells (NSCs), we analysed NSC populations at E16. Immunofluorescence showed increased SRY‐Box Transcription Factor 2‐positive (Sox2^+^) and paired box 6‐positive (Pax6^+^) radial glia in PD‐1^f/f; LysM‐Cre^ mice (Figure 5A–E), with elevated PH3^+^/Sox2^+^ cells in ventricular zones indicating enhanced proliferation (Figure 5C,D). In contrast, T box brain protein 2‐positive (Tbr2^+^) intermediate progenitors, Special AT‐Rich Sequence‐Binding Protein 2‐positive (Satb2^+^) upper‐layer neurons, and BCL11 Transcription Factor B‐positive (Ctip2^+^) lower‐layer neurons were unaffected (Figure 5F–J). These results demonstrate that myeloid PD‐1 deficiency specifically expands radial glia populations without altering neuronal production, suggesting PD‐1 signalling preferentially promotes NSC proliferation and astrocytic over neuronal differentiation.

### 3.6 NF‐κB Signalling Upregulates CXCL1 in PD‐1^f/f; LysM‐Cre^ Mice

**FIGURE 6:**
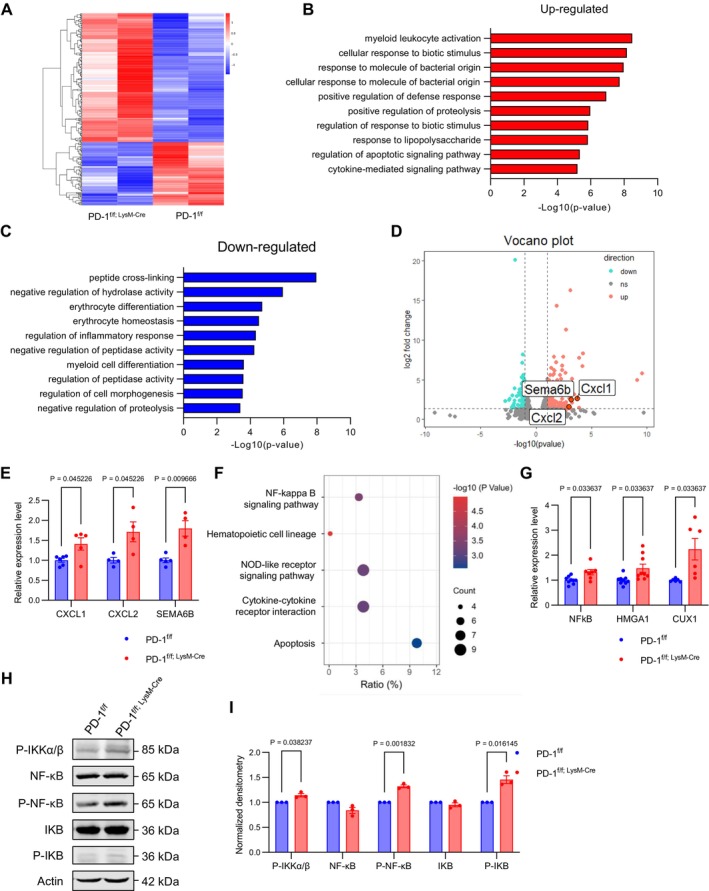
RNA‐seq analysis of the P3 fetal liver. (A) Heat map of RNA‐seq data from the P3 fetal liver of PD‐1^f/f^ mice and PD‐1^f/f; LysM‐Cre^ mice. (B and C) Gene ontology analysis of up‐regulated and down‐regulated genes associated with biological functions related to stimulating cell activation. (D) Volcano plots illustrating the down‐regulated (blue) and up‐regulated (red) genes in PD‐1^f/f; LysM‐Cre^ mice compared with the PD‐1^f/f^ mice. SEMA6B, CXCL1, and CXCL2 are labelled as differentially expressed genes. (E) Quantification of mRNA levels revealed increased expression of CXCL1, CXCL2, and SEMA6B in PD‐1^f/f; LysM‐Cre^ mice (*n* ≥ 4). (F) The KEGG pathway analysis focused on the NF‐κB signalling pathway. (G) Quantification of CXCL1‐related gene mRNA levels showed an increase in PD‐1^f/f; LysM‐Cre^ mice (*n* ≥ 6). (H) Western blot analysis of the expression levels of NF‐κB signalling pathway, Actin was detected as loading control. (I) Statistics showing an increased ratio of P‐IKKα/β, P‐NK‐κB, and P‐IKB in PD‐1^f/f; LysM‐Cre^ mice (*n* ≥ 3). Data are presented as mean ± SEM. Multiple t‐tests and nonparametric tests.

To investigate the internal mechanism by which myeloid‐specific PD‐1 ablation impacts astrocytes, we isolated CD11b^+^ myeloid cells from P3 fetal liver by flow cytometry to conduct RNA sequencing analysis. Our findings demonstrated that specific PD‐1 ablation alters gene expression in myeloid cells (Figure 6A). Comparative analysis of upregulated and downregulated genes revealed distinct biological processes with gene ontology analysis, indicating that many upregulated genes are associated with cell activation stimulation (Figure 6B,C). In this study, volcano maps were used to screen for differentially expressed genes in PD‐1^f/f; LysM‐Cre^ and PD‐1^f/f^ mice. The threshold was set as |log2FC| > 1 and *p* < 0.01, and significant up‐regulated genes (red) and down‐regulated genes (blue) were identified. Among them, the gene CXCL1 may be the key regulatory factor (Figure 6D). We confirmed that specific PD‐1 ablation increased the mRNA levels of CXCL1, CXCL2, and SEMA6B in myeloid cells (Figure 6E). We detected the expression of other genes (NF‐κB, HMGA1, CUX1) that synergistically interact with CXCL1, which also showed an increase (Figure 6G). To elucidate the production mechanism of CXCL1, we focused on the NF‐κB pathway using KEGG (Figure 6F). Additionally, expression levels of the NF‐κB signalling pathway, including phosphorylation molecules such as P‐IKKα/β, P‐NF‐κB, and P‐IKB, were elevated in PD‐1^f/f; LysM‐Cre^ mice (Figure 6H,I). These results suggest that CXCL1 expression is primarily driven by activation of the NF‐κB signalling pathway, correlating with the NF‐κB expression level.

### 3.7 CXCL1‐Driven JAK/STAT3 Signalling Pathway Regulates Astrogenesis

**FIGURE 7:**
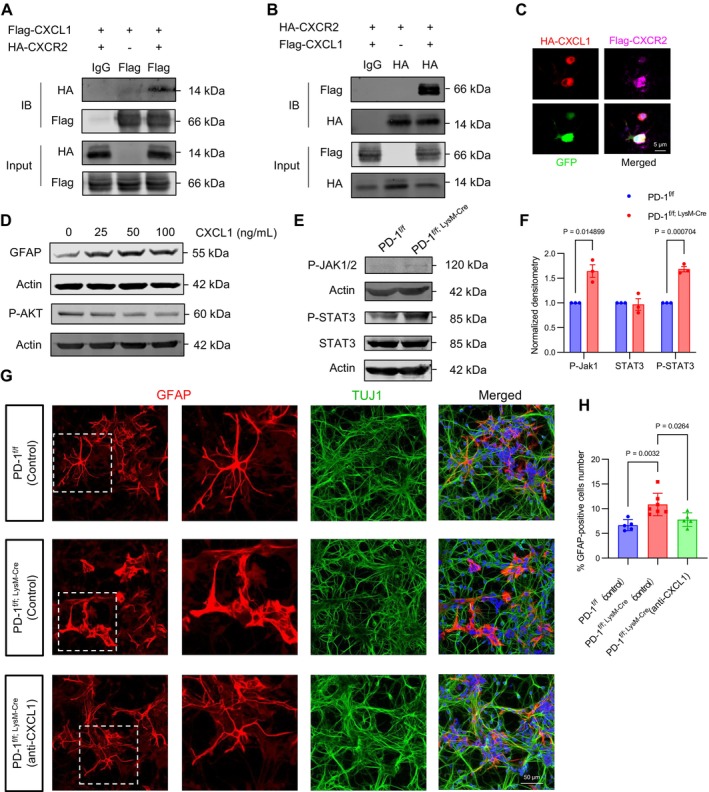
JAK/STAT3 pathway regulates the activation of astrocyte. (A and B) Co‐immunoprecipitation (CO‐IP) assays for the interaction between CXCL1 and CXCR2. (C) Confocal immunofluorescence image of FLAG and HA showing the colocalization of CXCL1 and CXCR2 (Scale bar, 5 μm). (D) Western blot analysis of the expression levels of the GFAP with different concentrations of CXCL1. (E) Western blot analysis of the expression levels of the JAK/STAT3 pathway, Actin was detected as loading control. (F) Statistics showing an increased ratio of P‐JAK1/2, and P‐STAT3 in PD‐1^f/f; LysM‐Cre^ mice (*n* = 3). (G) Confocal immunofluorescence image of TUJ1‐positive neurons and GFAP‐positive astrocytes from NSC (Scale bars, 50 μm). (H) Quantification of the percent of GFAP‐positive astrocytes showing decreased in NSC cocultured with anti‐CXCL1 in PD‐1^f/f; LysM‐Cre^ mice (*n* ≥ 5). Data are presented as mean ± SEM. Multiple t‐tests and nonparametric tests.

To investigate whether CXCL1 participate in a signalling pathway that regulates astrocytes through specific receptors such as CXCR2, which is expressed in astrocytes and can increase their numbers, we transfected 293FT cells with plasmids to obtain overexpressed CXCL1 and CXCR2 proteins, and then performed co‐immunoprecipitation. These assays demonstrated that Flag‐tagged CXCL1 effectively pulled down HA‐tagged CXCR2, and conversely, HA‐tagged CXCR2 pulled down Flag‐tagged CXCL1 (Figure 7A,B). Immunofluorescence staining further visualised this interaction, revealing that HA‐CXCL1 colocalized with Flag‐CXCR2 in the N2a cells (Figure 7C). To assess whether CXCL1 directly influences astrocyte proliferation, we cultured NSCs with various concentrations of CXCL1. Our results indicated that as CXCL1 concentration increased, the expression level of GFAP and phosphorylated AKT (P‐AKT) also increased (Figure 7D). The JAK/STAT3 pathway is known to promote astrocyte reactivity and activation. We found that expression level of phosphorylated JAK1/2 (P‐JAK1/2) and phosphorylated STAT3 (P‐STAT3) were elevated in PD‐1^f/f; LysM‐Cre^ mice (Figure 7E,F). These findings suggest that CXCL1 abundance may drive astrogenesis by engaging the JAK/STAT3 signalling pathway.

Furthermore, we utilised cocultured transwell systems to determine whether anti‐CXCL1 could rescue the abnormal astrocyte development in PD‐1^f/f; LysM‐Cre^ mice. Our results demonstrated that anti‐CXCL1 effectively reversed the increased number and the enlarged morphology of astrocytes in PD‐1^f/f; LysM‐Cre^ mice (Figure 7G,H). We conducted behavioural rescue experiments. After 5 days of intraperitoneal injection of inhibitors followed by behavioural experiments, PD‐1^f/f; LysM‐Cre^ mice returned to the activity level of PD‐1^f/f^ mice in various behavioural experiments (Figure S5).

## 4 Discussion

The CNS and the immune system, while distinct, communicate to maintain homeostasis. Microglia are derived from primitive haematopoietic stem cells. In the central nervous system, microglia exhibit different phenotypes and states depending on the environment, which can be either resting or active. Adult microglia are an independent self‐renewing population [19]. In inflammatory response, the release of pro‐inflammatory factors leads to changes in synapse‐related pathways such as postsynaptic N‐methyl‐D‐aspartic acid (NMDA) receptor activation and NF‐κB pathway inhibition. Thus, promoting or inhibiting the phagocytosis of microglia, leading to inappropriate synaptic pruning [20, 21]. Circulating myeloid cells may participate in astrocyte modulation, though their relative contribution requires further lineage‐tracing studies. There are still difficulties existing in tracking transient infiltration and resident microglial activity. Certain myeloid cells, such as dendritic cells and macrophages, reside within the CNS and contribute to T‐cell activation during EAE progression. Macrophages secrete signalling molecules that trigger inflammatory responses, influencing neighbouring cells and recruiting additional immune cells to help resolve inflammation [22, 23]. Accurate communication between CNS and myeloid cells is essential for proper immune function. In this study, we investigated the role of PD‐1 in myeloid cells during CNS development and identified the potential pathway through which PD‐1 functions as a key regulator of astrogenesis. While myeloid cells are known to provide innate immune defence against infection and injury, their functions within the CNS under steady‐state conditions still a lot unknown. These cells possess unique regulatory mechanisms that can influence innate immunity and may contribute to cognitive dysfunction [18]. Despite the growing recognition of the myeloid‐brain axis, studies in this area remain limited. As a prominent immune molecule, PD‐1 significantly impacts tumour development and is closely associated with the myeloid system [24]. Therefore, we aimed to investigate whether PD‐1 in the myeloid system affects brain development.

We revealed the crucial role of PD‐1 in myeloid cells in regulating astrocyte development during early cortical development. Our data suggest a potential trend of astrocyte activation, though further proliferation assays are needed to confirm this. PD‐1 expression increases rapidly as tumour size grows, triggering emergency myelopoiesis that activates T‐cells, which in return suppress antitumor activity [13, 25]. Our findings indicate that the number of PD‐1‐positive myeloid cells increase over time under physiological conditions. This suggests that these cells become more prevalent during development and may cover a broader area, playing a significant role in the immune response. In the context of inflammation, IFN‐γ and IL‐17A can synergize to enhance to Th1 (helper T cell 1) activation, while IL‐4 is produced by Th2 (helper T cell 1) cells. Modulating the inflammatory environment involves the upregulation and downregulation of various cytokines [26]. We examined cytokines such as IFN‐γ, IL‐4, and IL‐17A to assess the functional impact of PD‐1‐specific knockout in myeloid cells; however, no significant changes were observed. We suggest that ablation of PD‐1 does not alter cytokine release. Furthermore, the specific ablation of PD‐1 influenced the proliferation and modulation of proportions in myeloid cells. The increase in myeloid cells is attributed to a rise in GMPs, which subsequently leads to the differentiation of more granulocytes and monocytes.

Myeloid cells reprogram glucose metabolism to restore youthful immune function in brain disorders [10]. As key nutritional provider in the brain, astrocytes enhance their protein expression in response to PD‐1 signalling from myeloid cells. Throughout the various stages of astrocyte development, immunofluorescence staining with different astrocyte markers revealed that astrocytes‐related genes were highly expressed in PD‐1^f/f; LysM‐Cre^ mice. The hallmark of neuroprotective reactive phenotypes is increased astrocyte proliferation [27]. Astrocytes, as resident cells in the CNS, typically produce anti‐inflammatory factors in response to changes in the cellular environment, such as Heparin‐binding epidermal growth factor–like growth factor (HB‐EGF) [28]. N‐methyl‐D‐aspartate receptors (NMDAR) on astrocytes are sensitive to neuronal activity. Astrocytes enhance neural excitability by releasing glutamate and ATP, leading to increased excitation in mice [29, 30]. We found that PD‐1^f/f; LysM‐Cre^ mice exhibited more extroverted behaviour, reduced anxiety, and increased openness to exploration in behavioural experiments. Combined with an increase in astrocytes, these behavioural changes may be underlying alterations in the expression of brain‐derived neurotrophic factor (BDNF) in the brain's reward region [31]. Knockout mice modulate neural adaptations in the reward circuitry of the mouse brain, resulting in significant positive effects on behaviour [32]. Astrocytes regulate the activity of adjacent glutamatergic neurons by releasing D‐serine, which leads to neuronal excitation [33].

To elucidate the mechanisms by which PD‐1 regulates functions in myeloid cells, we performed transcriptome sequencing on both specific knockout mice and wild‐type mice. This approach enabled us to cluster biological processes and identify downstream with greater precision. Our analysis revealed that CXCL1 is a key downstream target, proposed to be linked to the SOX4 promoter, activating its transcription through the NF‐κB pathway [34]. An increase in NF‐κB levels is associated with elevated pro‐inflammatory cytokines and chemokines, including CXCL1. The activation of the NF‐κB‐dependent CXCL1/CXCR2 signalling pathway are implicated in modulating pain‐related neuronal responses [35, 36]. The release and binding of CXCL1 to the CXCR2 receptor in astrocytes regulated their development via the JAK/STAT3 signalling pathway. The release and binding of CXCL1 to the CXCR2 receptor in astrocytes triggered their proliferation via the JAK/STAT3 signalling pathway. This pathway, which regulates astrocyte reactivity, represents an important therapeutic target in neurological disorders [37, 38]. Notably, the increase in CXCL1 levels in myeloid cells correlates with a rise in astrocyte numbers. Abnormalities in CXCL1 have been shown to trigger astrogliosis [39]. Astrocytes also serve as paracrine mediators, enhancing synaptic connections and boosting neuronal firing [40]. We hypothesize that this phenomenon may be intrinsic to more active and extroverted mice; however, many aspects of this hypothesis remain to be confirmed. And further mechanistic studies (e.g., CXCR2 knockout models or ligand‐receptor blocking assays) would strengthen this causal inference.

The findings not only demonstrate the critical regulatory role of PD‐1 in myeloid cells on astrocytes but also provide a theoretical framework and direction for exploring the myeloid‐brain axis. The brain is not an immunologically privileged site; peripheral immune cells can influence brain function, potentially leading to behavioural and cognitive deficits [21]. Subtle changes during fetal development and the early life stages may significantly impact immune system development, highlighting the importance of fetal health during this critical period. A deeper understanding of neuroimmune interactions will enable us to formulate effective strategies to mitigate neuronal and cognitive impairment associated with innate immune disorders.

## Author Contributions

**Jie Qin:** conceptualization; methodology; investigation; writing – original draft; writing – review and editing. **Chong Wang:** investigation. **Sihan Li:** investigation; visualisation. **Yanyan Wang:** writing – review and editing. **Tingting He:** investigation. **Jianwei Jiao:** funding acquisition. **Fen Ji:** supervision; project administration; writing – review and editing.

## Ethics Statement

All mice studies supervised and approved by Institutional Animal Care and Use Committee of Institute of Zoology, Chinese Academy of Sciences (IOZ‐IACUC‐2020‐025). All animal housing and experiments followed the institutional guidelines for the care and use of laboratory animals.

## Conflicts of Interest

The authors declare no conflicts of interest.

## Supporting information


**Figure S1.** Specific ablation PD‐1 in myeloid cells. (A) Schematic diagram of *Pd1* & LysM‐Cre knockout identification results. (B) The knockdown efficiency was verified at the RNA level (*n* = 3). (C) Western blot analysis of the expression levels of PD‐1 in bone marrow in PD‐1^f/f; LysM‐Cre^ mice and PD‐1^f/f^ mice. (D) Western blot analysis of the expression levels of CD68 and CX3CR1 in PD‐1^f/f; LysM‐Cre^ mice and PD‐1^f/f^ mice. (E) Statistics showing unchanged expression levels of CD68 and CX3CR1 in brain tissue between PD‐1^f/f; LysM‐Cre^ mice and PD‐1^f/f^ mice (*n* = 3). (F) Statistics showing expression levels of PD‐1 decreased in PD‐1^f/f; LysM‐Cre^ mice (*n* = 3). (G) Confocal immunofluorescence image of Iba1 and Cre in cerebral cortex (Scale bars, 50 μm). (H) Statistics showing the proportion of Cre^+^Iba1^+^ and Iba1^+^ cells (*n* = 3). (I) Confocal immunofluorescence image of NeuN and Cre in cerebral cortex (Scale bars, 50 μm). (J) Statistics showing the proportion of Cre^+^NeuN^+^ and NeuN^+^ cells (*n* = 3). (K) Confocal immunofluorescence image of PD‐1 and CD11b in pia mater (Scale bars, 5 μm). Data are presented as mean ± SEM. Multiple t‐tests and nonparametric tests.
**Figure S2.** Flow cytometry in PD‐1 in PD‐1^f/f; LysM‐Cre^ mice and PD‐1^f/f^ mice. (A) The schema represented the sequential steps of the gating strategy. The various cell populations within the bone marrow were depicted. (B) FACS histograms and contour plots depicting the percentage of positive cells and bar graphs. (C) Representative FACS analysis plots for CD11b^+^F4/80^+^, CD11b^+^Ly6C^+^, and CD11b^+^Ly6G^+^ ratio in the fetal liver. (D) Statistics showing the number of flow sorting in CD11b^+^F4/80^+^, CD11b^+^Ly6C^+^, and CD11b^+^Ly6G^+^ per 100,000 cells (*n* ≥ 6). (E) Statistics showing decreased F4/80^+^ and Ly6G^+^ and increased Ly6C^+^ in PD‐1^f/f; LysM‐Cre^ mice (*n* ≥ 6). (F) Statistics showing unchanged expression levels of cytokines between PD‐1^f/f; LysM‐Cre^ mice and PD‐1^f/f^ mice (*n* ≥ 6). Data are presented as mean ± SEM. One‐way ANOVA and multiple t‐tests and nonparametric tests.
**Figure S3.** Specific ablation of PD‐1 in myeloid cells leads to persistent increased astrocyte production and enlarged morphology. (A and C) Confocal immunofluorescence image showed GFAP^+^ astrocytes per square millimetre at P3 and P8 (Scale bars, 200 μm). (B and D) Quantification of the increase in the number of GFAP^+^ astrocytes in PD‐1^f/f; LysM‐Cre^ mice (*n* = 3). (E) Confocal immunofluorescence image showed S100β^+^ astrocytes per square millimetre at P18 (Scale bars, 200 μm). (F) Quantification of the increase in the number of S100β^+^ astrocytes in PD‐1^f/f; LysM‐Cre^ mice (*n* = 3). (G) Confocal immunofluorescence image showed Olig2^+^ oligodendrocytes per square millimetre (Scale bars, 200 μm). (H) Quantification of the unchanged number of Olig2^+^ oligodendrocytes in PD‐1^f/f; LysM‐Cre^ mice (*n* = 3). (I) Confocal immunofluorescence image showed PDGFRα^+^ oligodendrocytes per square millimetre (Scale bars, 200 μm). (J) Quantification of the unchanged number of PDGFRα^+^ oligodendrocytes in PD‐1^f/f; LysM‐Cre^ mice (*n* = 3). Data are presented as mean ± SEM. Multiple t‐tests and nonparametric tests.
**Figure S4.** The modality and number of astrocytes changed in co‐culture. (A) Confocal immunofluorescence image showed PH3^+^GFAP^+^ astrocytes per square millimetre at P0 (Scale bars, 100 μm). (B) Quantification of the increase in the number of PH3^+^GFAP^+^ astrocytes in PD‐1^f/f; LysM‐Cre^ mice (*n* ≥ 3). (C) The diagram of myeloid cells and human NPCs coculture system. (D) Confocal immunofluorescence image of GFAP^+^ astrocytes derived from NSCs (Scale bars, 10 μm). (E) Quantification of the percent of GFAP^+^ astrocytes showing increased number and enlarged astrocyte form in NSCs cocultured in PD‐1^f/f; LysM‐Cre^ mice in E16 (*n* ≥ 3). (F) Quantification of the percent of GFAP^+^ astrocytes showing increased number and enlarged astrocyte form in NSCs cocultured in PD‐1^f/f; LysM‐Cre^ mice in P3 (*n* ≥ 3). Data are presented as mean ± SEM. Multiple t‐tests and nonparametric tests.
**Figure S5.** Behavioural rescue experiment in mice. (A and B) The time spent in the center area and outside in open field. (*n* ≥ 9). (C and D) The discrimination score and discrimination ratio were higher in PD‐1^f/f; LysM‐Cre^ mice in the novel object recognition test (*n* ≥ 9). (E–G) The time spent in closed arms (E) and open arms (F) and center (G) in the elevated plus‐maze test (*n* ≥ 8). (H) PD‐1^f/f; LysM‐Cre^ mice spent significantly more time in the novel arm compared to controls (*n* ≥ 8). (I and J) Social affiliation and sociability duration (I) in the sociability session and social memory and novelty duration (J) in the social novelty preference session in the three‐chamber social interaction test (*n* ≥ 8). Data are presented as mean ± SEM. One‐way ANOVA and multiple t‐tests and nonparametric tests.

**Table S1.** List of primary antibodies.
**Table S2.** List of secondary antibodies.
**Table S3.** List of flow antibodies.
**Table S4.** List of major primers.

## Data Availability

The accession number for the RNA‐sequencing data reported in this article is GSE277873.
