# Supplementary material for: Myeloid PD‐1 Regulates Astrocyte Development and Leads to Active Behaviours
Source: Cell Prolif. 2025 Jun 29;59(2):e70082. doi: 10.1111/cpr.70082 (PMC12877958; doi:10.1111/cpr.70082)
Supplement: Supplementary file 1 — Figure S1. Specific ablation PD‐1 in myeloid cells. (A) Schematic diagram of Pd1 & LysM‐Cre knockout identification results. (B) The knockdown efficiency was verified at the RNA level (n = 3). (C) Western blot analysis of the expression levels of PD‐1 in bone marrow in PD‐1f/f; LysM‐Cre mice and PD‐1f/f mice. (D) Western blot analysis of the expression levels of CD68 and CX3CR1 in PD‐1f/f; LysM‐Cre mice and PD‐1f/f mice. (E) Statistics showing unchanged expression levels of CD68 and CX3CR1 in brain tissue between PD‐1f/f; LysM‐Cre mice and PD‐1f/f mice (n = 3). (F) Statistics showing expression levels of PD‐1 decreased in PD‐1f/f; LysM‐Cre mice (n = 3). (G) Confocal immunofluorescence image of Iba1 and Cre in cerebral cortex (Scale bars, 50 μm). (H) Statistics showing the proportion of Cre+Iba1+ and Iba1+ cells (n = 3). (I) Confocal immunofluorescence image of NeuN and Cre in cerebral cortex (Scale bars, 50 μm). (J) Statistics showing the proportion of Cre+NeuN+ and NeuN+ cells (n = 3). (K) Confocal immunofluorescence image of PD‐1 and CD11b in pia mater (Scale bars, 5 μm). Data are presented as mean ± SEM. Multiple t‐tests and nonparametric tests. Figure S2. Flow cytometry in PD‐1 in PD‐1f/f; LysM‐Cre mice and PD‐1f/f mice. (A) The schema represented the sequential steps of the gating strategy. The various cell populations within the bone marrow were depicted. (B) FACS histograms and contour plots depicting the percentage of positive cells and bar graphs. (C) Representative FACS analysis plots for CD11b+F4/80+, CD11b+Ly6C+, and CD11b+Ly6G+ ratio in the fetal liver. (D) Statistics showing the number of flow sorting in CD11b+F4/80+, CD11b+Ly6C+, and CD11b+Ly6G+ per 100,000 cells (n ≥ 6). (E) Statistics showing decreased F4/80+ and Ly6G+ and increased Ly6C+ in PD‐1f/f; LysM‐Cre mice (n ≥ 6). (F) Statistics showing unchanged expression levels of cytokines between PD‐1f/f; LysM‐Cre mice and PD‐1f/f mice (n ≥ 6). Data are presented as mean ± SEM. One‐way ANOVA and multip [file CPR-59-e70082-s002.pdf]

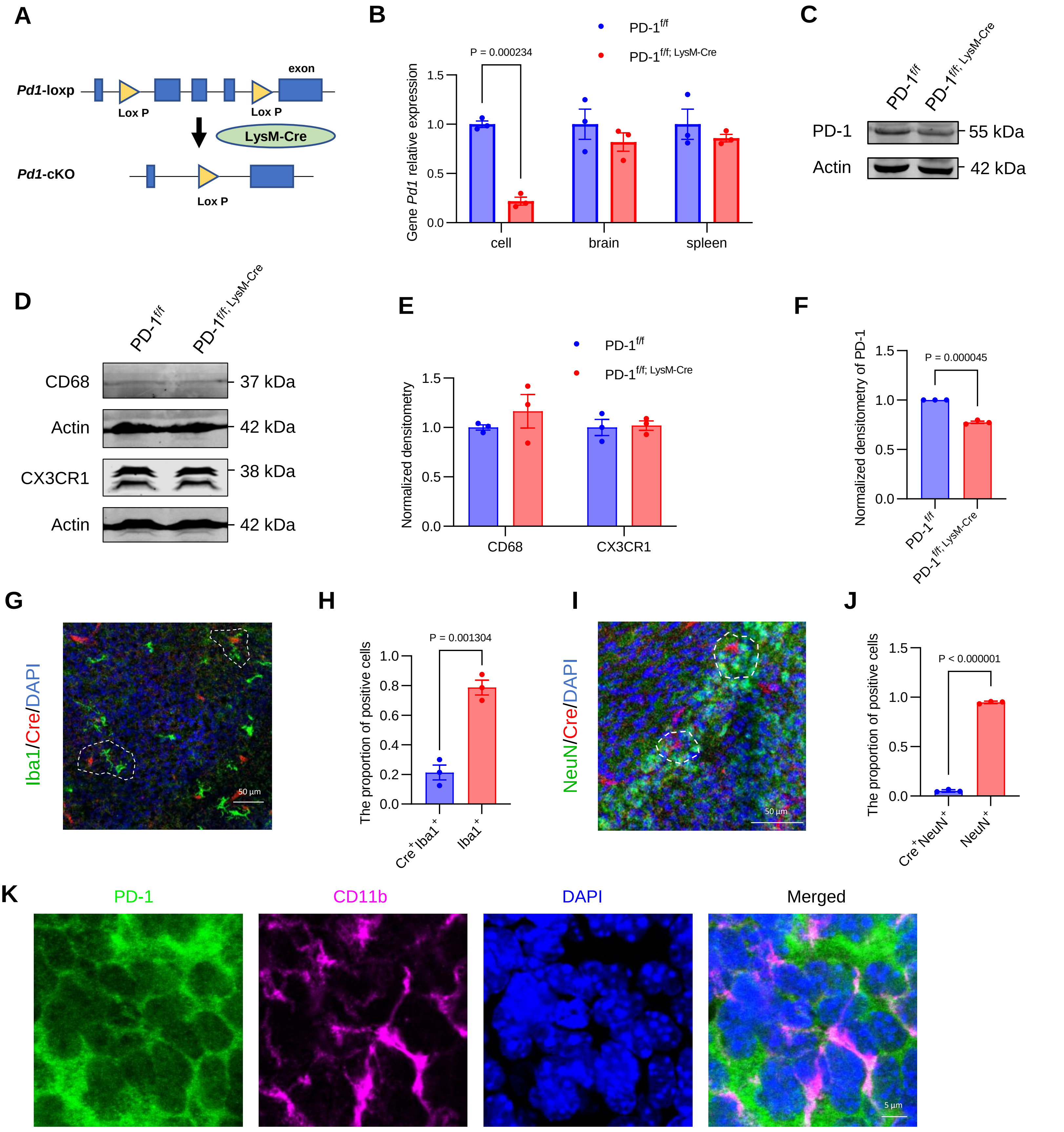

A
Pd1-loxp
exon
Lox P
Lox P
LysM-Cre
Pd1-cKO
Lox P
B
PD-1 f/f
PD-1 f/f; LysM-Cre
P = 0.000234
Gene Pd1 relative expression
1.5
1.0
0.5
0.0
cell
brain
spleen
C
PD-1 f/f
PD-1 f/f; LysM-Cre
PD-1
55 kDa
Actin
42 kDa
D
PD-1 f/f
PD-1 f/f; LysM-Cre
CD68
37 kDa
Actin
42 kDa
CX3CR1
38 kDa
Actin
42 kDa
E
PD-1 f/f
PD-1 f/f; LysM-Cre
Normalized densitometry
1.5
1.0
0.5
0.0
CD68
CX3CR1
F
P = 0.000045
Normalized densitometry of PD-1
1.5
1.0
0.5
0.0
PD-1 f/f
PD-1 f/f; LysM-Cre
G
Iba1/Cre/DAPI
50 µm
H
P = 0.001304
The proportion of positive cells
1.0
0.8
0.6
0.4
0.2
0.0
Cre+Iba1+
Iba1+
I
NeuN/Cre/DAPI
50 µm
J
P < 0.000001
The proportion of positive cells
1.5
1.0
0.5
0.0
Cre+NeuN+
NeuN+
K
PD-1
CD11b
DAPI
Merged
5 µm

Supplemental Figure 1. Specific ablation PD-1 in myeloid cells.

(A) Schematic diagram of *Pd1* & LysM-Cre knockout identification results.

(B) The knockdown efficiency was verified at the RNA level (n = 3).

(C) Western blot analysis of the expression levels of PD-1 in bone marrow in PD-$1^{f/f;\ LysM\text{-}Cre}$ mice and PD-$1^{f/f}$ mice.

(D) Western blot analysis of the expression levels of CD68 and CX3CR1 in PD-$1^{f/f;\ LysM\text{-}Cre}$ mice and PD-$1^{f/f}$ mice.

(E) Statistics showing unchanged expression levels of CD68 and CX3CR1 in brain tissue between PD-$1^{f/f;\ LysM\text{-}Cre}$ mice and PD-$1^{f/f}$ mice (n = 3).

(F) Statistics showing expression levels of PD-1 decreased in PD-$1^{f/f;\ LysM\text{-}Cre}$ mice (n = 3).

(G) Confocal immunofluorescence image of Iba1 and Cre in cerebral cortex (Scale bars, 50μm).

(H) Statistics showing the proportion of $Cre^{+}Iba1^{+}$ and $Iba1^{+}$ cells (n = 3).

(I) Confocal immunofluorescence image of NeuN and Cre in cerebral cortex (Scale bars, 50 μm).

(J) Statistics showing the proportion of $Cre^{+}NeuN^{+}$ and $NeuN^{+}$ cells (n = 3).

(K) Confocal immunofluorescence image of PD-1 and CD11b in pia mater (Scale bars, 5 μm).

Data are presented as mean ± SEM. Multiple t-tests and nonparametric tests.

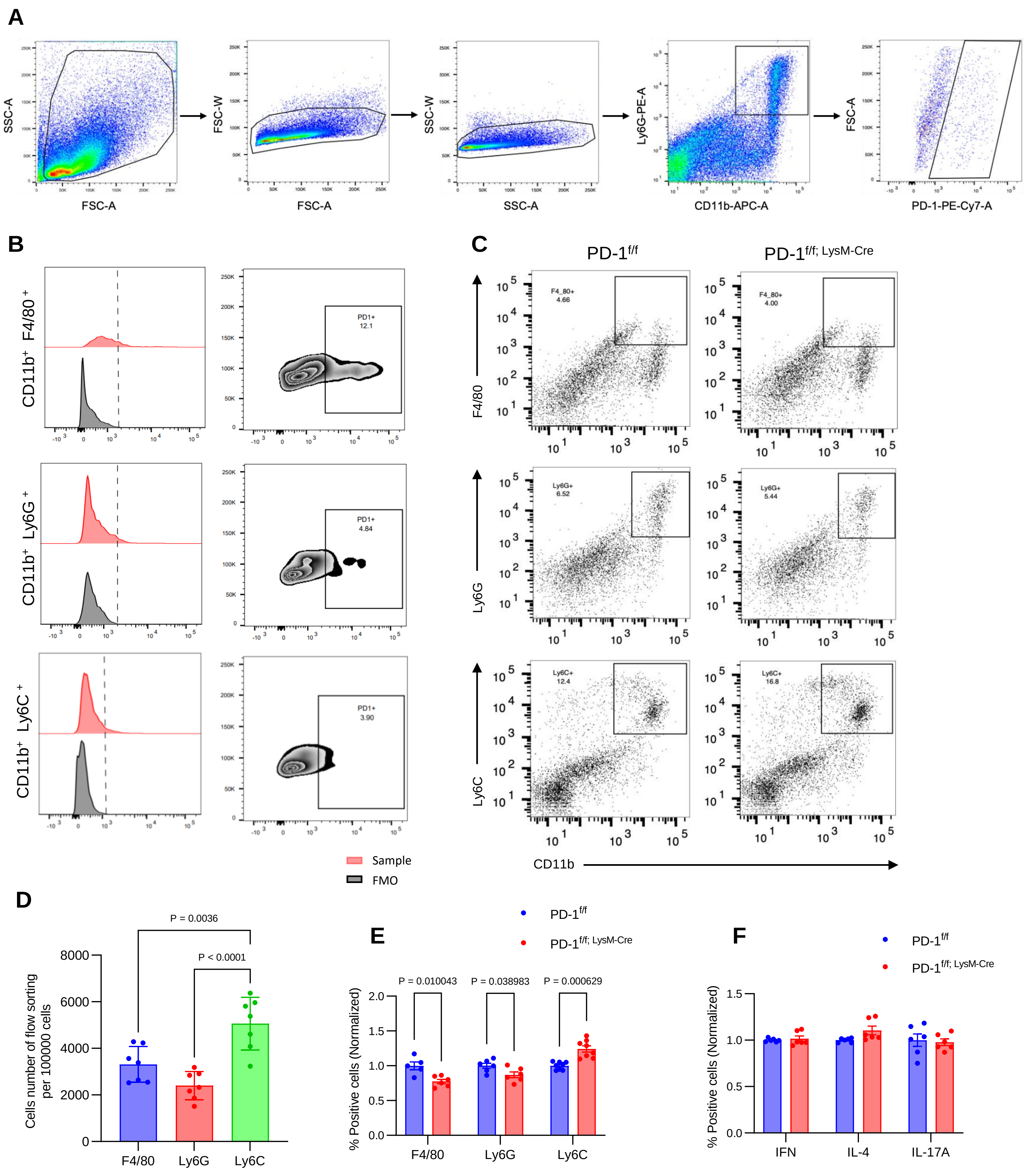
A
SSC-A
FSC-A
FSC-W
FSC-A
SSC-W
SSC-A
Ly6G-PE-A
CD11b-APC-A
FSC-A
PD-1-PE-Cy7-A
B
CD11b+ F4/80+
PD1+ 12.1
CD11b+ Ly6G+
PD1+ 4.84
CD11b+ Ly6C+
PD1+ 3.90
Sample
FMO
C
PD-1f/f
PD-1f/f; LysM-Cre
F4/80
F4_80+ 4.66
F4_80+ 4.00
Ly6G
Ly6G+ 6.52
Ly6G+ 5.44
Ly6C
Ly6C+ 12.4
Ly6C+ 16.8
CD11b
D
P = 0.0036
P < 0.0001
Cells number of flow sorting per 100000 cells
F4/80
Ly6G
Ly6C
E
PD-1f/f
PD-1f/f; LysM-Cre
P = 0.010043
P = 0.038983
P = 0.000629
% Positive cells (Normalized)
F4/80
Ly6G
Ly6C
F
PD-1f/f
PD-1f/f; LysM-Cre
% Positive cells (Normalized)
IFN
IL-4
IL-17A

Supplemental Figure 2. Flow cytometry in PD-1 in PD-1$^{f/f; LysM-Cre}$ mice and PD-1$^{f/f}$ mice.
(A) The schema represented the sequential steps of the gating strategy. The various cell populations within the bone marrow were depicted.
(B) FACS histograms and contour plots depicting the percentage of positive cells and bar graphs.
(C) Representative FACS analysis plots for CD11b$^+$F4/80$^+$, CD11b$^+$Ly6C$^+$, and CD11b$^+$Ly6G$^+$ ratio in the fetal liver.
(D) Statistics showing the number of flow sorting in CD11b$^+$F4/80$^+$, CD11b$^+$Ly6C$^+$, and CD11b$^+$Ly6G$^+$ per 100000 cells ($n \geq 6$).
(E) Statistics showing decreased F4/80$^+$ and Ly6G$^+$ and increased Ly6C$^+$ in PD-1$^{f/f; LysM-Cre}$ mice ($n \geq 6$).
(F) Statistics showing unchanged expression levels of cytokines between PD-1$^{f/f; LysM-Cre}$ mice and PD-1$^{f/f}$ mice ($n \geq 6$).
Data are presented as mean ± SEM. One-way ANOVA and multiple t-tests and nonparametric tests.

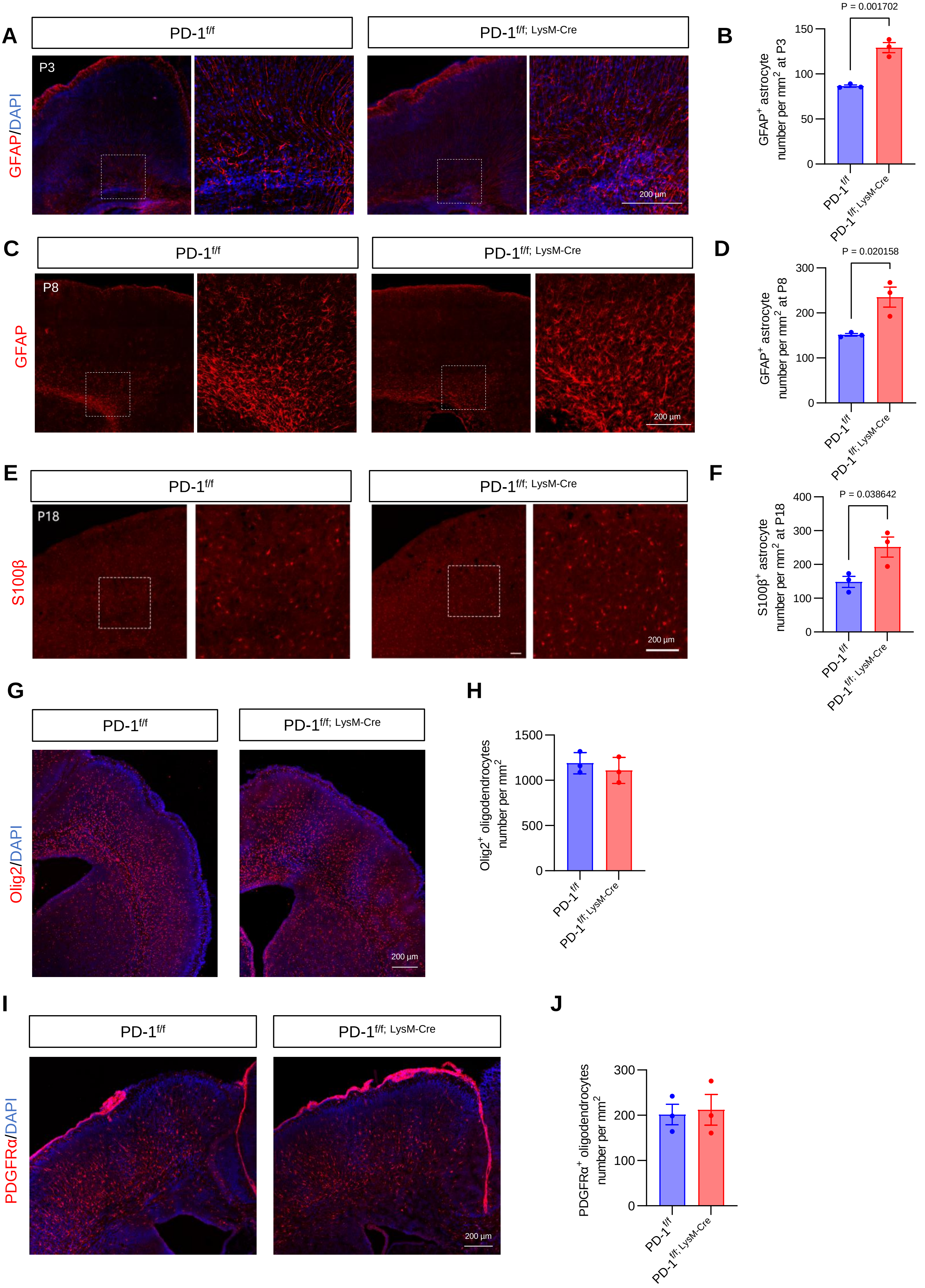

A
PD-1f/f
PD-1f/f; LysM-Cre
P3
GFAP/DAPI
200 µm
B
P = 0.001702
GFAP+ astrocyte number per mm2 at P3
150
100
50
0
PD-1f/f
PD-1f/f; LysM-Cre
C
PD-1f/f
PD-1f/f; LysM-Cre
P8
GFAP
200 µm
D
P = 0.020158
GFAP+ astrocyte number per mm2 at P8
300
200
100
0
PD-1f/f
PD-1f/f; LysM-Cre
E
PD-1f/f
PD-1f/f; LysM-Cre
P18
S100β
200 µm
F
P = 0.038642
S100β+ astrocyte number per mm2 at P18
400
300
200
100
0
PD-1f/f
PD-1f/f; LysM-Cre
G
PD-1f/f
PD-1f/f; LysM-Cre
Olig2/DAPI
200 µm
H
Olig2+ oligodendrocytes number per mm2
1500
1000
500
0
PD-1f/f
PD-1f/f; LysM-Cre
I
PD-1f/f
PD-1f/f; LysM-Cre
PDGFRα/DAPI
200 µm
J
PDGFRα+ oligodendrocytes number per mm2
300
200
100
0
PD-1f/f
PD-1f/f; LysM-Cre

Supplemental Figure 3. Specific ablation of PD-1 in myeloid cells leads to persistent increased astrocyte production and enlarged morphology.
(A and C) Confocal immunofluorescence image showed $GFAP^+$ astrocytes per square millimeter at P3 and P8 (Scale bars, 200 μm).
(B and D) Quantification of the increase in the number of $GFAP^+$ astrocytes in PD-$1^{f/f; LysM-Cre}$ mice (n = 3).
(E) Confocal immunofluorescence image showed $S100\beta^+$ astrocytes per square millimeter at P18 (Scale bars, 200 μm).
(F) Quantification of the increase in the number of $S100\beta^+$ astrocytes in PD-$1^{f/f; LysM-Cre}$ mice (n = 3).
(G) Confocal immunofluorescence image showed $Olig2^+$ oligodendrocytes per square millimeter (Scale bars, 200 μm).
(H) Quantification of the unchanged number of $Olig2^+$ oligodendrocytes in PD-$1^{f/f; LysM-Cre}$ mice (n = 3).
(I) Confocal immunofluorescence image showed $PDGFR\alpha^+$ oligodendrocytes per square millimeter (Scale bars, 200 μm).
(J) Quantification of the unchanged number of $PDGFR\alpha^+$ oligodendrocytes in PD-$1^{f/f; LysM-Cre}$ mice (n = 3).
Data are presented as mean ± SEM. Multiple t-tests and nonparametric tests.

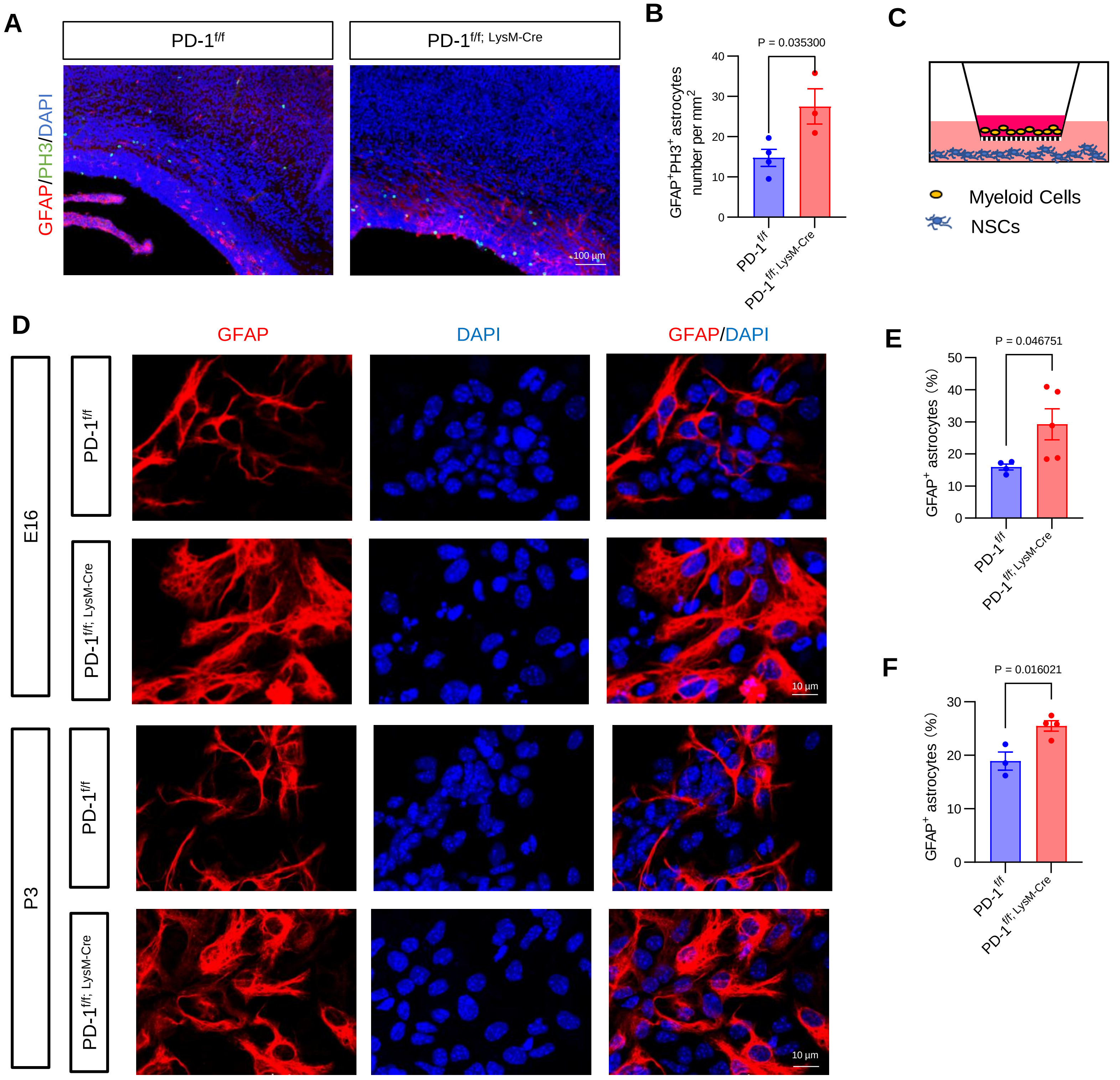
A
PD-1f/f
PD-1f/f; LysM-Cre
GFAP/PH3/DAPI
100 µm
B
P = 0.035300
GFAP+PH3+ astrocytes number per mm2
PD-1f/f
PD-1f/f; LysM-Cre
C
Myeloid Cells
NSCs
D
GFAP
DAPI
GFAP/DAPI
E16
P3
10 µm
E
P = 0.046751
GFAP+ astrocytes (%)
F
P = 0.016021
GFAP+ astrocytes (%)

Supplemental Figure 4. The modality and number of astrocytes changed in co-culture.

(A) Confocal immunofluorescence image showed $PH3^{+}GFAP^{+}$ astrocytes per square millimeter at P0 (Scale bars, 100 μm).

(B) Quantification of the increase in the number of $PH3^{+}GFAP^{+}$ astrocytes in PD-$1^{f/f;\ LysM\text{-}Cre}$ mice ($n \geq 3$).

(C) The diagram of myeloid cells and human NPCs coculture system.

(D) Confocal immunofluorescence image of $GFAP^{+}$ astrocytes derived from NSCs (Scale bars, 10 μm).

(E and F) Quantification of the percent of GFAP-positive astrocytes showing increased number and enlarged astrocyte form in NSCs cocultured in PD-$1^{f/f;\ LysM\text{-}Cre}$ mice ($n \geq 3$).

Data are presented as mean ± SEM. Multiple t-tests and nonparametric tests.

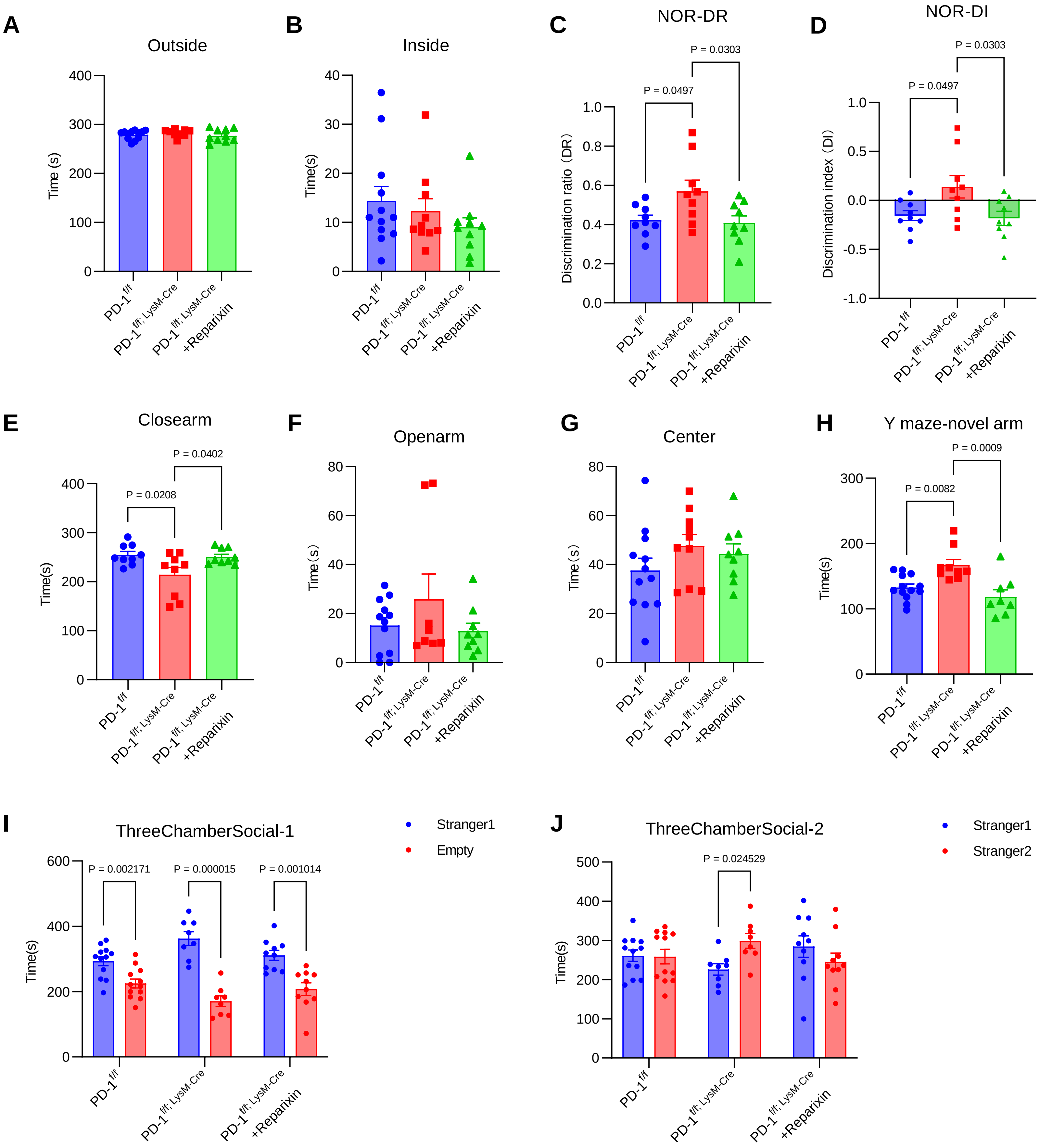
A
Outside
Time (s)
B
Inside
Time(s)
C
NOR-DR
P = 0.0497
P = 0.0303
Discrimination ratio（DR）
D
NOR-DI
P = 0.0497
P = 0.0303
Discrimination index（DI）
E
Closearm
P = 0.0208
P = 0.0402
Time(s)
F
Openarm
Time (s)
G
Center
Time (s)
H
Y maze-novel arm
P = 0.0082
P = 0.0009
Time(s)
I
ThreeChamberSocial-1
Stranger1
Empty
P = 0.002171
P = 0.000015
P = 0.001014
Time(s)
J
ThreeChamberSocial-2
Stranger1
Stranger2
P = 0.024529
Time(s)
PD-1 f/f
PD-1 f/f; LysM-Cre
PD-1 f/f; LysM-Cre +Reparixin

Supplemental Figure 5. Behavioral rescue experiment in mice.
(A and B) The time spent in the center area and outside in open field. ($n \geq 9$).
(C and D) The discrimination score and discrimination ratio were higher in PD-1$^{f/f;\ LysM\text{-}Cre}$ mice in the novel object recognition test ($n \geq 9$).
(E-G) The time spent in closed arms (E) and open arms (F) and center (G) in the elevated plus-maze test ($n \geq 8$).
(H) PD-1$^{f/f;\ LysM\text{-}Cre}$ mice spent significantly more time in the novel arm compared to controls ($n \geq 8$).
(I and J) Social affiliation and sociability duration (I) in the sociability session and social memory and novelty duration (J) in the social novelty preference session in the three-chamber social interaction test ($n \geq 8$).
Data are presented as mean ± SEM. One-way ANOVA and multiple t-tests and nonparametric tests.
