# Supplementary material for: Myeloid PD‐1 Regulates Astrocyte Development and Leads to Active Behaviours
Source: Cell Prolif. 2025 Jun 29;59(2):e70082. doi: 10.1111/cpr.70082 (PMC12877958; doi:10.1111/cpr.70082)
Supplement: Supplementary file 2 — Table S1. List of primary antibodies. Table S2. List of secondary antibodies. Table S3. List of flow antibodies. Table S4. List of major primers. [file CPR-59-e70082-s001.pdf]

The primary antibodies required in this study are shown in the table below.

Supplementary Table 1. List of primary antibodies

Antibody	Source	Catalog
PD-1	Rabbit	Proteintech 18106-1-AP
PD-1	Rabbit	Abcam ab214421
CD11b	Mouse	Bio-Rad MCA711G
BrdU	Rat	Abcam ab6326
Ki67	Rabbit	Abcam ab15580
ALDH1L1	Mouse	Abcam ab56777
NeuN	Mouse	Millipore mab377
GFAP	Rabbit	Dako Z0334
GFAP	Rabbit	Sigma G6171
S100β	Rabbit	Abcam ab52642
β-actin	Mouse	Proteintech 66009-1-Ig
β-actin	Rabbit	Proteintech 20536-1-AP
BLBP	Rabbit	Abcam ab32423
GS	Mouse	Millipore MAB302
Sox2	Rabbit	CST 3728S
PH3	Rabbit	CST 3377S
Pax6	Rabbit	Millipore AB2237
Tbr2	Rabbit	Abcam ab23345
Satb2	Mouse	Abcam ab51502
Ctip2	Rat	Abcam ab18465
P-IKKα/β	Rabbit	CST 2697
NF-κB	Mouse	CST 6956
P-NF-κB	Rabbit	CST 3033
IKB	Rabbit	Abmart T55026
P-IKB	Rabbit	Abmart TP56280
Flag	Mouse	Sigma F1804
HA	Rabbit	CST 3724S
P-AKT	Rabbit	CST 3787S
P-JAK1/2	Mouse	CST 66245S
P-STAT3	Rabbit	Abmart T56566
STAT3	Mouse	CST 9139
Pax6	Mouse	DSHB AB528427
Sox2	Goat	R&D AF2018

The secondary antibodies required in this study are shown in the table below.

Supplementary Table 2. List of secondary antibodies

Antibody	Catalog
Cy3-Donkey anti-rabbit IgG (H+L)	Jackson 711-165-152
Cy3-Donkey Anti-mouse IgG (H+L)	Jackson 715-165-150
488-Donkey anti-rabbit IgG (H+L)	Jackson 711-546-152
Cy3-Donkey anti-rat IgG (H+L)	Jackson 712-165-150
Donkey anti-Rabbit 800	LICOR 926-32213
Donkey anti-mouse 680	LICOR 926-68072
488-Donkey anti-mouse IgG (H+L)	Invitrogen A21202

The flow antibodies required in this study are shown in the table below.

Supplementary Table 3. List of flow antibodies

Antibody	Catalog
APC anti-mouse/human CD11b Antibody	Biolegend 101212
FITC anti-mouse F4/80 Antibody	Biolegend 123107
PE anti-mouse Ly-6G Antibody	Biolegend 127608
CD45 Monoclonal Antibody, PE	Invitrogen 12045182
Alexa Fluor 488 anti-mouse Ly-6C Antibody	Biolegend 128022
PE/Cyanine7 anti-mouse PD-1 Antibody	Biolegend 109109
PD-L1 Monoclonal Antibody, PE-Cyanine7	eBioscience 25598280
PE-Cyanine7 IL-4 Monoclonal Antibody	Invitrogen, 25-7041-80
PE-eFluor 610 IL-17A Monoclonal Antibody	Invitrogen, 61-7177-80
PE IFN gamma Monoclonal Antibody	Invitrogen, 12-7311-82

The major primers required in this study are shown in the table below.

Supplementary Table 4. List of major primers

Primer	Sequence
LysM-Cre Mutant	CCCAGAAATGCCAGATTACG
LysM-Cre Common	CTTGGGCTGCCAGAATTTCTC
LysM-Cre Wild type	TTACAGTCGGCCAGGCTGAC
actin-F	GGCTGTATTCCCCTCCATCG
actin-R	CCAGTTGGTAACAATGCCATGT
PD1-Loxp-F	CTGGAACATCTTGAACAGGAGTG
PD1-Loxp-R	TGTGAATAAGTAACCTCCAACAAGC
PD1-RT-F	CTGAAAAACAGGCCGCCTTC
PD1-RT-R	ATGGCCCCACAGAGGTAGAT
Cxcl1-F	ACTGCACCCAAACCGAAGTC
Cxcl1-R	TGGGGACACCTTTTAGCATCTT
Cxcl2-F	CCAACCACCAGGCTACAGG
Cxcl2-R	GCGTCACACTCAAGCTCTG
Sema6b-F	ACTTGAGCCACTATCCCGTGT
Sema6b-R	TGGTACAGGTTATCTCTGTCCC
NF-κB-p65-F	GCGAGAGGAGCACAGATACC
NF-κB-p65-R	AGGGGTTGTTGTTGGTCTGG
HMGA1-F	GGTCGGGAGTCAGAAAGAGC
HMGA1-R	ATTCTTGCTTCCCTTTGGTCG
CUX1-F	TGATGCCACCGCAACAGTATT
CUX1-R	GCGTAAATCCTCTGGAGTGTTCT
